# Examining the Role of Mobile Phone and Social Media Use in Predicting Psychological Distress Among Young Adults: A Cross-Sectional Study

**DOI:** 10.7759/cureus.110232

**Published:** 2026-06-04

**Authors:** Anamika Sahu, Ankita Dhiman, Manoj Sharma

**Affiliations:** 1 Clinical Psychology, National Institute of Mental Health and Neurosciences, Bengaluru, IND

**Keywords:** anxiety, depression, problematic smartphone use, problematic social media use, stress, young adults

## Abstract

Background

Problematic smartphone use (PSU) and problematic social media use (PSMU) have been associated with adverse mental health outcomes, yet they are often examined independently. This study examines PSU and PSMU as predictors of depression, anxiety, and stress among Indian youth.

Methods

A cross-sectional design was employed using standardized self-report measures assessing PSU, PSMU, and psychological distress among 779 participants aged 18-25 years. Hierarchical multiple regression analyses were conducted to assess the predictive value of age, gender, problematic mobile phone use, and social media use, as well as their unique contributions.

Results

PSMU significantly predicted depression (R² = 0.112, F(4, 774) = 24.327, p < 0.001), anxiety (R² = 0.090, F(4, 774) = 19.236, p < 0.001), and stress (R² = 0.112, F(4, 774) = 24.500, p < 0.001), whereas PSU did not emerge as a significant predictor of psychological distress. Younger age and female gender were also associated with higher psychological distress, suggesting potential vulnerability factors.

Conclusions

PSMU emerged as a significant predictor of depression, anxiety, and stress, whereas PSU did not independently predict psychological distress after controlling for age and gender.

## Introduction

Access to smartphones and the internet has become an integral part of everyday life. In India, a recent press release stated that 85.5% of households possess at least one smartphone and that 86.3% of households have access to the internet within the household premises [1]. Coupled with the relatively low cost of mobile data, this has contributed to a rapid increase in smartphone and social media use in recent years. While such widespread access offers several benefits, emerging evidence suggests that excessive and problematic patterns of use may be associated with adverse health and mental health outcomes [2]. In India, the prevalence of smartphone addiction has been reported to range from 25.2% to 66% [2,3], while 39% of individuals have been found to exhibit problematic smartphone use (PSU) [4]. Furthermore, excessive internet use for social media activities is highly prevalent among youth, with one study reporting that 76.3% demonstrated problematic social media use (PSMU) [5].

Both PSU and PSMU have been linked to negative mental health outcomes. Excessive smartphone use has been negatively associated with psychological well-being, depression, anxiety, insomnia, and life satisfaction [2,4]. Additionally, PSMU has been associated with depression, anxiety, and stress [6,7]. However, findings across studies remain inconsistent, with some reporting weak or non-significant associations [8].

In the Indian context, contemporary youth represent one of the earliest cohorts to have experienced widespread smartphone access during their formative developmental years. This period, encompassing late adolescence and emerging adulthood, is also marked by increased vulnerability to psychological concerns such as depression, anxiety, and stress [9]. Furthermore, PSU and PSMU are often examined in isolation, making it difficult to understand how they may differentially relate to mental health outcomes when considered together. Taken together, these gaps highlight the need to examine the role of both PSU and PSMU in relation to mental health outcomes within this population. Accordingly, the present study examines PSU and PSMU as predictors of depression, anxiety, and stress among Indian youth.

## Materials and methods

This is a cross-sectional study. Colleges, workplaces, and other community settings in Bengaluru, Karnataka, were approached for data collection.

Ethical considerations

The study was approved by the Institutional Ethics Committee (No. NIMHANS/35th IEC (BEH.SC.DIV.)/2022), and informed consent was obtained from colleges, workplaces, and individual participants prior to administration of the survey tools. All data were collected anonymously and treated confidentially.

Participants

A nonprobability convenience sampling method was used to recruit individuals aged 18-25 years over a period of one year, based on the specified inclusion and exclusion criteria. The inclusion criteria included individuals who had been using the internet or mobile devices for a minimum period of one year. Individuals who were unwilling to participate were excluded from the study. Based on a prevalence of 6% for technology addiction in Bangalore, with a 95% confidence level and a relative error of 10%, the minimum required sample size was calculated to be 450. After accounting for an anticipated non-response rate of approximately 20%, the total sample size targeted for the study was 750 participants. The final sample of 779 participants exceeded the required sample size, ensuring adequate statistical power to detect meaningful associations.

Assessment tools

Semi-structured Data Sheet

It was designed to collect participants’ sociodemographic and mobile and internet use information (Appendix 1).

Problematic Mobile Phone Use Questionnaire (PMPUQ)

Problematic mobile phone use was assessed using the PMPUQ [10]. It consists of 30 items rated on a four-point Likert scale and assesses four dimensions: prohibited use, dangerous use, dependence, and financial problems. Total scores are obtained by summing the item responses, with higher scores indicating greater problematic mobile phone use.

Bergen Social Media Addiction Scale (BSMAS)

Social media addiction was assessed using the BSMAS [11]. It has six items, where higher scores indicate a greater risk of addiction to social media. It demonstrated good test-retest reliability (r = 0.82).

Depression, Anxiety, and Stress Scale (DASS-21)

DASS-21 is a self-report measure designed to assess depression, anxiety, and stress in non-clinical populations [12]. The scale contains 21 items rated on a four-point Likert scale, with each of the three subscales comprising seven items. Subscale scores are calculated by summing the relevant items.

Procedure

Eligible individuals were invited to participate after the study’s purpose and procedures were explained. Informed consent was obtained from the participants. They completed demographic questions, followed by the study tools. Assessments were conducted offline using self-report measures in English, which took approximately 20-30 minutes to complete. The instruments were administered in either individual or group settings. A minimum of three attempts were made to obtain complete responses before participants were classified as dropouts and excluded from the final analyses.

Statistical analysis

Descriptive statistics were used for the study variables, using frequency, percentage, mean, and standard deviation. The internal consistency of the study measures was assessed using Cronbach's alpha. The BSMAS demonstrated moderate reliability (α = 0.617, 95% CI 0.573, 0.658), whereas the PMPUQ showed good reliability (α = 0.859, 95% CI 0.805, 0.897). The DASS-21 subscales also exhibited good internal consistency, with alpha coefficients of 0.804 (95% CI 0.717, 0.883) for Anxiety, 0.864 (95% CI 0.793, 0.904) for Depression, and 0.816 (95% CI 0.744, 0.880) for Stress. Overall, the measures demonstrated acceptable to good reliability, supporting their use in subsequent analyses.

A hierarchical multiple linear regression analysis was used for three reasons: (a) to examine the overall prediction of depression, anxiety, and stress based on problematic mobile phone use, social media use, age, and gender; (b) to determine the unique contribution of each set of variables through sequential entry into the model; and (c) to provide a more nuanced understanding of the relative roles of problematic mobile phone use and PSMU in explaining psychological distress.

Prior to regression analysis, the assumptions of normality, homoscedasticity, and multicollinearity were assessed and found to be satisfactory, with no evidence of multicollinearity (variance inflation factor (VIF) < 5; tolerance > 0.20). A p-value of less than 0.05 was considered statistically significant.

## Results

A total of 850 individuals were approached for the study. Of these, 779 provided consent and completed the assessment and were therefore included in the final analysis. The mean age of participants was 20.93 (SD = 3.08) years; the majority were female (n = 484; 62.13%), single (n = 688; 88.32%), pursuing an undergraduate academic program (n = 495; 63.54%), and belonged to a nuclear family (n = 540; 69.32%) and middle socioeconomic strata (n = 779; 100%).

Table 1 shows smartphone-use-related variables. The majority of the participants had a smartphone (85.75%). More than half of the participants checked their phone as the first activity in the morning (53.79%). They used their phone more than intended (41.46%), felt bad when their phone was not nearby (60.20%), and experienced vibration of the mobile even when there were no calls or text messages (8.08%). Many of the participants increased their phone use when they experienced boredom (57.89%) or loneliness (75.87%). On average, participants spent 2 hours 50 minutes per day on the internet, engaging in activities such as browsing, social media use, watching videos, gaming, shopping, and downloading applications. Of this total time, they spent an average of 75.15 minutes per day on social media.

**Table 1:** Variables related to smartphone and social media use (n = 779)

Variables		Value
Type of phone	Smartphone	668 (85.75)
	Basic phone	94 (12.07)
	Both	17 (2.18)
	Cell-compatible watch	4 (0.51)
First thing in the morning is checking the phone	Yes	419 (53.79)
	No	360 (46.21)
Using the phone more than supposed to use	Yes	323 (41.46)
	No	456 (58.54)
Feel bad when the phone is not near	Yes	469 (60.20)
	No	310 (39.80)
Feel the vibration of the mobile, even though there is no call/text message?	<5 times a day	621 (79.72)
	6-10 times a day	95 (12.20)
	>10 times a day	63 (8.08)
Start using the phone when feeling bored	Yes	451 (57.89)
	No	328 (42.11)
Start using the phone when feeling lonely	Yes	591 (75.87)
	No	188 (24.13)

The mean score on the PMPUQ was 73.79 (SD = 16.39), indicating moderately high problematic use of mobile phones. The BSMAS mean score was 13.04 (SD = 5.44), indicating low risk of social media addiction. DASS-21 total scores ranged from 0 to 104 (mean ± SD = 23.92 ± 23.9). The depression domain mean score (mean ± SD = 8.34 ± 9.59) was higher than the mean score for stress (mean ± SD = 8.26 ± 8.44) and anxiety (mean ± SD = 7.38 ± 7.88). Depressive symptoms were reported by 36.07% of participants, anxiety symptoms by 42.36%, and stress by 18.74% of participants. Notably, a small proportion experienced extremely severe depression (4.2%), anxiety (10.8%), and stress (0.9%).

Table 2 presents the results of the hierarchical regression analysis examining the predictors of depression, anxiety, and stress. In Model 1 for depression, depression was significantly predicted by age and gender (R² = 0.029, F(2, 776) = 11.676, p < 0.001), and explained 2.9% of the variance in depression, with gender as a significant predictor (B = -2.336, p = 0.001). The addition of PMPUQ in Model 2 did not significantly improve the model (ΔR² = 0.000, p = 0.530). In the final model, the inclusion of social media use (Model 3) significantly improved the model (ΔR² = 0.082, p < 0.001), with the model explaining 11.2% of the variance in depression (R² = 0.112, F(4, 774) = 24.327, p < 0.001).

**Table 2:** Hierarchical multiple regression predicting depression, anxiety, and stress among mobile phone and social media users Note: N = 779; * p < 0.05; ** p < 0.01; *** p < 0.001. PMPUQ: Problematic Mobile Phone Use Questionnaire; SMU: Social Media Use

Variables		Model 1			Model 2			Model 3		
		B	SE B	β	B	SE B	β	B	SE B	β
Depression	Constant	19.206***	2.471	-	20.118***	2.866	-	12.758**	2.879	-
	Gender	-2.336**	0.708	-	-2.311**	0.710	-	-1.971**	0.681	-
	Age	-0.450***	0.112	-0.144	-0.448***	0.112	-0.144	-0.336**	0.108	-0.108
	PMPUQ	-	-	-	-0.013	0.021	-0.022	-0.006	0.020	-0.011
	SMU	-	-	-	-	-	-	1.593***	0.189	0.289
	R^2^	0.029	-	-	0.030	-	-	0.112	-	-
	F	11.676***	-	-	7.910***	-	-	24.327***	-	-
	ΔR^2^	0.029	-	-	0.000	-	-	0.082	-	-
	ΔF	11.676***	-	-	0.396	-	-	71.423***	-	-
Anxiety	Constant	14.571***	2.036	-	15.164***	2.362	-	9.706***	2.393	-
	Gender	-2.024***	0.584	-	-2.008***	0.585	-	-1.756**	0.566	-
	Age	-0.284**	0.092	0.111	-0.283**	0.092	0.111	-0.199*	0.090	-0.078
	PMPUQ	-	-	-	-0.008	0.017	-0.018	-0.003	0.017	-0.007
	SMU	-	-	-	-	-	-	1.181***	0.157	0.261
	R^2^	0.023	-	-	0.024	-	-	0.090	-	-
	F	9.255***	-	-	6.246***	-	-	19.236***	-	-
	ΔR^2^	0.023	-	-	0.000	-	-	0.067	-	-
	ΔF	9.255***	-	-	0.247	-	-	56.856***	-	-
Stress	Constant	12.679***	2.193	-	13.386***	2.544	-	6.279	2.533	-
	Gender	-1.924**	0.629	-	-1.905**	0.630**		-1.577	0.599	-
	Age	-0.154	0.099	-0.056	-0.153	0.099	-0.056	-0.044**	0.095	-0.016
	PMPUQ	-	-	-	-0.010	0.018	-0.020	-0.003	0.017	-0.007
	SMU	-	-	-	-	-	-	1.538***	0.166	0.317
	R^2^	0.013	-	-	0.014	-	-	0.112	-	-
	F	5.250**	-	-	3.598*	-	-	24.500***	-	-
	ΔR^2^	0.013	-	-	0.000	-	-	0.099	-	-
	ΔF	5.250*	-	-	0.303	-	-	86.022***	-	-

In Anxiety Model 1, age and gender significantly predicted anxiety (R² = 0.023, F(2, 776) = 9.255, p < 0.001), explaining 2.3% of the variance, with gender as a significant predictor (B = -2.024, p < 0.001). The addition of PMPUQ in Model 2 did not significantly improve the anxiety model (ΔR² = 0.000, p = 0.620). In contrast, the inclusion of social media use in the final model (Model 3) showed a substantial improvement (ΔR² = 0.090, p < 0.001), with the model explaining 9.0% of the variance in anxiety (R² = 0.090, F(4, 774) = 19.236, p < 0.001).

The first model of stress showed that age and gender significantly predicted stress (R² = 0.013, F(2, 776) = 5.250, p = 0.005), but explained only a small proportion of variance (1.3%), with gender as a significant predictor (B = -1.924, p = 0.002). The addition of PMPUQ in Model 2 did not significantly improve the model (ΔR² = 0.000, p = 0.582). However, the inclusion of social media use in the final model (Model 3) led to a substantial improvement (ΔR² = 0.099, p < 0.001), with the model explaining 11.2% of the variance in stress (R² = 0.112, F(4, 774) = 24.500, p < 0.001).

## Discussion

This study of young adults found that social media use significantly predicted depression, anxiety, and stress, whereas problematic mobile phone use did not predict these psychological outcomes. These findings are consistent with earlier research demonstrating significant associations between social media use and psychological distress [6,13,14]. The present study results extend existing evidence by indicating that social media use is independently associated with higher levels of depression, anxiety, and stress, even after accounting for demographic factors. Younger age and female gender may represent additional vulnerabilities. One possible explanation is that excessive social media use may displace meaningful daily activities, reduce face-to-face interactions, limit physical activity, and disrupt sleep, thereby increasing psychological distress.

The study findings may also be influenced by the higher proportion of females in the sample. Generally, women are more likely to experience depression and anxiety, possibly due to biological, psychological, and social factors [15]. Although the participants in the present study spent relatively less time on the internet and social media compared to previous research [16], it is the quality of social media engagement that appears to be more relevant than frequency of use. Some patterns increase distress, whereas perceived online social support may be protective [17,18].

This relationship is complex and does not appear to be straightforward. Excessive social media use may contribute to increased levels of depression, anxiety, and stress, while individuals experiencing loneliness, low self-esteem, anhedonia, or anxiety may turn to social media as a coping strategy, possibly exacerbating their symptoms [19,20]. The cognitive-behavioural model of addiction emphasizes that pre-existing psychopathology (i.e., distal factors) and maladaptive cognitions (i.e., proximal factors) interact to develop and sustain problematic use of social media [21]. In other words, psychosocial difficulties may predispose individuals to develop maladaptive cognitions, leading to impaired self-regulation and, consequently, negative outcomes associated with social media use. Greater emotional investment in social media and heightened concerns about self-presentation may increase psychological distress [22,23], such as through upward social comparison, particularly on visually oriented platforms like Instagram [24,25]. Hence, PSMU may shape how individuals internalize online experiences, with factors such as rumination and low self-esteem increasing vulnerability to depression [26].

In the present study, most participants had access to smartphones, reported checking them first thing in the morning, and felt uncomfortable when the phone was not nearby, suggesting a degree of psychological reliance on smartphones. Additionally, a notable proportion of participants reported using their smartphones more than intended, particularly during periods of boredom and loneliness, suggesting possible difficulties in regulating smartphone use that may be influenced by situational emotional states. However, problematic mobile phone use did not significantly predict depression, anxiety, or stress. Previous research has also shown mixed findings, with some studies reporting only weak associations with psychological distress [27]. One possible explanation is the relatively lower levels of problematic mobile phone use and psychological distress in the current sample, which may have limited the detection of significant associations. Additionally, problematic mobile phone use may not capture emotionally salient aspects of digital engagement, such as social comparison or self-evaluation, that are more directly related to psychological distress [22-25]. These findings suggest that clinicians and policymakers should adopt a more nuanced approach to prevention programs by emphasizing digital literacy, healthy social media habits, sleep hygiene, and emotional resilience instead of relying only on screen-time reduction strategies or complete bans on phone or online platform use.

Furthermore, the findings should be interpreted in light of the study’s strengths and limitations.

This study focused on young adults aged 18-25 years and involved the simultaneous assessment of social media use, problematic mobile phone use, and psychological outcomes. However, the cross-sectional design limits conclusions regarding causal directionality, indicating the need for longitudinal research. Additionally, the use of convenience sampling and the predominance of female, unmarried, and middle-socioeconomic-status participants in the sample may have introduced sampling-related bias and may limit the representativeness and generalizability of findings to the broader population. Reliance on self-report measures may also have introduced response bias.

## Conclusions

In conclusion, PSMU significantly predicted depression, anxiety, and stress among young adults, whereas problematic mobile phone use did not, suggesting that patterns of digital engagement may be more relevant to psychological well-being than overall mobile phone use. Younger age and female gender also emerged as potential vulnerabilities. As this relationship may be bidirectional, longitudinal and experimental studies are needed to clarify directionality. These findings highlight the importance of interventions promoting healthy social media use, digital literacy, and emotional resilience among young adults.

## Appendices

Appendix 1

**Table 3:** Semi-structured data collection sheet

Semi-structured data collection sheet				
1	Age (years):			
2	Gender	M:		
					F:		
		3	Socioeconomic status	Rs…………………… / month			
							(Per-Capita Monthly Family Income)			
4	Education									
						5					Occupation			
															6	Marital status	· Single:		
· Married: (yrs)																			
· Divorced: (yrs)																
· Widowed: (yrs)																
7	Family status:	· Nuclear														
		· Joint		
		· Single parent		
					8	How many cell phones do you own?			
9	Which type of mobile you are using	· Smartphone:							
		· Basic phone							
		· Both		
		· Cell compatible watch		
					10	As soon as you get up in the morning, the first thing you do is to check your phone	· Yes		
· No						
11	Are you using technology (both internet & cell phone) more than you supposed to use?	· Yes		
		· No		
12	If you don’t have your smart phone, do you feel bad?	· Yes		
		· No		
	How many times in a day do you feel vibration of mobile, even though there is no call/text message?	<5 times a day		
13		6-10 times a day		
		>10 times a day.		
14	When you feel bored, do you start browsing your Smartphone?	· Yes		
		· No		
15	When you feel lonely, do you use your smart phone?	· Yes		
		· No		
16	Time spent using phone per day (approx):			
		minute	hrs	how many times in a day
	· Texting:			
	· Internet:			
	· shopping			
	· Gaming			
	· Pornography			
	· Listening to music			
	· App downloads			
	· Calling only in emergencies.			
	· Texting & calling both			
	· Taking pictures & selfie.			
	· Watching videos			
	· Browsing			
	· Productive app			
	· Other use:			
